# Maximality and ontology: how axiom content varies across philosophical frameworks

**DOI:** 10.1007/s11229-017-1336-9

**Published:** 2017-03-04

**Authors:** Neil Barton, Sy-David Friedman

**Affiliations:** Kurt Gödel Research Center for Mathematical Logic (KGRC), University of Vienna, Währinger Straße, 25, 1090 Vienna, Austria

**Keywords:** Set theory, Foundations of mathematics, Philosophy of mathematics, Multiversism

## Abstract

Discussion of new axioms for set theory has often focused on conceptions of *maximality*, and how these might relate to the iterative conception of set. This paper provides critical appraisal of how certain maximality axioms behave on different conceptions of ontology concerning the iterative conception. In particular, we argue that forms of multiversism (the view that any universe of a certain kind can be extended) and actualism (the view that there are universes that cannot be extended in particular ways) face complementary problems. The latter view is unable to use maximality axioms that make use of *extensions*, where the former has to contend with the existence of extensions violating maximality axioms. An analysis of two kinds of multiversism, a Zermelian form and Skolemite form, leads to the conclusion that the *kind* of maximality captured by an axiom differs substantially according to background ontology.

## Introduction

The philosophical and mathematical development of set theory and its philosophy has been shaped by (at least) two different phenomena: paradox and independence. The former afflicted early naive attempts to axiomatise a theory of reified collections, and the latter remains a pervasive phenomenon in set-theoretic practice.

These two aspects have both led scholars to question whether or not there is a single ‘absolute’ universe of sets. On the side of paradox, given any particular universe $\mathcal {V}$, there are conditions $\phi (x)$ such that for every set *y* in $\mathcal {V}$, either $\phi (y)$ or $\lnot \phi (y)$, yet there is no set of all objects satisfying $\phi (x)$. This is conceptually puzzling; given the thought that all that one must do to characterise a set is provide its membership conditions, such a condition $\phi (x)$*prima facie* provides the resources to do just that. Hellman expresses the problem as follows:

“Consider the predicate “is a set” or “is an ordinal”. In our overall semantics, we naturally wish to assign an extension to such predicates. But, on the standard platonist picture, such extensions would be proper classes. (Of course, they cannot be consistently treated as “sets” in the technical sense; but they would be recognized as totalities of some sort, and this is enough to generate the predicament just described.) It is worth attempting to develop an alternative picture.” (Hellman 1989, p. 55)

The predicament Hellman describes needs a little more explanation to make the point clear. A natural thought concerning sets is that all that one need do in order to define a set is provide a precise determination of an extension. Such a determination provides us with the membership conditions of the set to be defined. Linnebo generalises this thought from the (merely first-order definable) conditions Hellman considers, to (possibly arbitrary) instances of plural reference and quantification^1^:

“We can thus give a complete and precise characterization of the set that *xx* would form if they did form a set. What more could be needed for such a set to exist?” (Linnebo 2010, p. 146)

This kind of thought will, of course, be anathema to anyone who holds that there is a definite height to the set-theoretic hierarchy.^2^ However, if one *is* moved by the thought that all we need to do to produce a set is determine a precise extension, then one way of avoiding this predicament is to allow that there is no *absolute* universe of sets, but rather that any universe may be extended (in a manner we make precise later). This would then allow the puzzling ‘proper classes’ of one universe to be sets in an extended universe. Continuing with Hellman, he writes:

“Every structure...has a proper extension, both in the sense of inclusion and in the sense that it, or some copy, occurs as a “member” of its proper extensions (i.e. in the domain of the relevant membership relation).” (Hellman 1989, p. 59)

Thus, viewing the sequence of set-theoretic structures as unbounded and always extendible provides the resources to have those things that satisfy $\phi (x)$ within some universe form a legitimate set in an extended structure.^3^

The methods employed in showing the independence results have also motivated the idea that any universe is extendible. The standard way of showing a sentence $\psi$ to be independent of $\mathbf {ZFC}$ is to construct a model of $\mathbf {ZFC}$ where $\psi$ holds (thereby showing that, if $\mathbf {ZFC}$ is consistent, then so is $\mathbf {ZFC} + \psi$), and also construct a model where $\lnot \psi$ holds (thereby showing that $\psi$ is not provable, if $\mathbf {ZFC}$ is consistent). Often, these models are very natural: for example in a forcing construction, if the first model is transitive and well-founded, then so is the extension. Thus, in proving various independence results, we construct a vast ‘zoo’ of different epistemic^4^ set-theoretic possibilities. Some have taken this as evidence for the claim that there is no ‘absolute’ inextensible universe of sets. Hamkins, for example, writes:

“This abundance of set-theoretic possibilities poses a serious difficulty for the universe view, for if one holds that there is a single absolute background concept of set, then one must explain or explain away as imaginary all of the alternative universes that set theorists seem to have constructed. This seems a difficult task, for we have a robust experience in those worlds, and they appear fully set theoretic to us.” (Hamkins 2012, p. 418)

While the philosophical attitudes to the seriousness of this difficulty vary^5^ a multiversism about set theory offers an elegant interpretation of discourse involving outer models and use of the symbol ‘*V*’. Instead of having to view these possibilities as illusory, we might instead take them to be indicative of modal relations between many universes. The various set-theoretic constructions exhibiting independence are then to be viewed as providing ways of moving among different universes accessible from one another.

Despite pervasive independence in set theory, there are those that hold that the truth-values of many sentences are discoverable through the addition of well-motivated additions to the axioms of $\mathbf {ZFC}$. A champion of this cause was Gödel, who wrote the following concerning certain large cardinal axioms:

“These axioms show clearly, not only that the axiomatic system of set theory as used today is incomplete, but also that it can be supplemented without arbitrariness by new axioms which only unfold the content of the concept of set explained above.” (Gödel 1964, pp. 260–261)

Of course, it is one thing to discuss possible axiomatic extensions of $\mathbf {ZFC}$, and quite another to provide cogent philosophical arguments to persuade the philosophico-mathematical community to accept these additions. While set theorists will likely continue to work with and study multiple different incompatible axiom systems, the possibility remains open to argue that certain axioms extending $\mathbf {ZFC}$ may nonetheless be part of (or at least harmonise well with) our set concept, and thus that some extension of $\mathbf {ZFC}$ should replace $\mathbf {ZFC}$ itself as our ‘canonical’ theory of sets.^6^ One seemingly attractive line has been the study of principles that try to capture *maximality* in set theory.^7^ We want (so the thinking goes) the set-theoretic structures with which we work to be as rich as possible, with as many and varied sets as possible. In a footnote to the second version of his seminal paper on the Continuum Hypothesis, Gödel writes:

“On the other hand, from an axiom in some sense opposite to this one,^8^ the negation of Cantor’s conjecture could perhaps be derived. I am thinking of an axiom which (similar to Hilbert’s completeness axiom in geometry) would state some maximum property of the system of all sets, whereas axiom *A* [i.e. $V=L$] states a minimum property. Note that only a maximum property would seem to harmonize with the concept of set...” (Gödel 1964, pp. 262–263, footnote 23)

We see here Gödel looking to intuitions concerning maximality in a search for a resolution of *CH*. Since Gödel’s paper, there have been several programmes that attempt to combine notions of maximality with our concept of set in order to explore the space of epistemic possibilities in searching for resolution of independence.^9^ This paper explores philosophical issues surrounding the development of maximality and how it relates to different varieties of multiversism. In particular, we will argue that the flavour of multiversism chosen affects the kind of maximality appealed to. Our strategy is as follows:

After these initial remarks, we first (§1) lay out some conceptual preliminaries. We briefly outline the iterative conception of set, and explain how it relates to debates concerning actualism and multiversism in set theory. We present what some have regarded as a promising line of inquiry in the search for new axioms: the consideration of maximality criteria. We then (§2) explain the use of extensions in formulating notions of maximality, and note that different kinds of multiversism and actualism face complementary problems; for the latter extensions are not available whereas the former has to contend with the fact that many universes exhibiting maximality have extensions which fail to satisfy maximality axioms. Next (§3) we provide responses on behalf of two different combinations of multiversism and actualism. We argue that given this analysis, the kind of maximality captured by a particular axiom is radically dependent upon the relevant philosophical backdrop. Finally (§4) we conclude that this is a feature of axiomatisation in set theory that ought to be borne in mind when formulating and justifying new axioms for set theory. In addition, some technical details are provided in an Appendix (§5).

## Actualism, multiversism, and the iterative conception

Before continuing further, we should be precise about the senses in which we will be using the terms ‘Actualism’ and ‘Multiversism’, and lay down some conceptual preliminaries.

### The iterative conception of set

Firstly, we shall be clear about the concept of set with which we work (the so called ‘iterative conception’ of set), especially as it is useful in providing explanation of different species of multiversism. Under the iterative conception, we iterate the power set operation along the sequence of ordinals, starting with the empty set^10^ and taking unions at limits. More formally, using transfinite recursion, we define ‘the’ iterative hierarchy *V*, comprised of the stages $V_\alpha$, as follows:

$$V_0 = \emptyset $$.

$V_{\alpha +1} = \mathcal {P}(V_\alpha )$, for successor ordinal $(\alpha +1)$.

$$V_\lambda = \bigcup _{\beta < \lambda } V_\beta $$, for limit $$\lambda $$.

$$V = \bigcup _{\alpha \in On} V_\alpha $$.

The iterative conception has a number of pleasing features. This is not least because it motivates a restriction on the comprehension schema; in a particular universe we should not expect there to be a set of all the *x* such that $\phi (x)$ holds for any condition whatsoever. In particular, conditions such as ‘*x* is an ordinal’, ‘$x \not \in x$’, and ‘*x* is a set’ have sets satisfying them unboundedly in any iterative structure of the above form, and so we should not expect there to be a set of all *x* such that $\phi (x)$ within a universe.

A second reason that many have been attracted to the iterative conception is that one can provide motivations for the axioms of $\mathbf {ZFC}$ based on iterative notions. Various attempts have been given in this regard, for example Boolos (1971). The extent to which these motivations are satisfactory is a controversial issue,^11^ and we will not concern ourselves directly with the justification of $\mathbf {ZFC}$ on the basis of the iterative conception. For now, we merely note that the iterative conception is at least amenable to the provision of heuristic motivations for the $\mathbf {ZFC}$ axioms.

For our purposes, the key facet of working within the iterative conception of set is that it provides a framework in which we can be more specific about the kinds of multiversism we envisage. In particular, the distinction between issues of *height* (i.e. the length of the iteration of the $V_\alpha$) and *width* (i.e. what subsets exist at successor stages) will be key for being precise about different kinds of multiversism.

### Actualism and multiversism

Once we are working within the iterative conception of set, we should be attentive as to how (from a philosophical and conceptual perspective) the truth values of set-theoretic sentences are settled. Since sets belong to stages obtained by iterating the powerset operation through the ordinals, the truth-value of a set-theoretic statement depends on two crucial parameters:

By *questions of height* we mean questions concerning what ordinals exist to index the $V_\alpha$.

By *questions of width* we mean questions concerning what subsets of $V_\alpha$ are contained in $V_{\alpha +1}$.

Once one has established what height a particular hierarchy has and the nature of its powerset operation, then one will have settled all truth values for set-theoretic statements within the structure. However, the extent to which one views questions of height and width as receiving an actualist or multiversist answer will affect what truth values one is prepared to ascribe to set-theoretic sentences.

We can come to an understanding of the differences between different kinds of actualism and multiversism by examining attitudes concerning what is guaranteed by the iterative conception. First, however, we require a remark concerning what we hope to achieve with the iterative conception. There are some philosophers, a good example being Hamkins (2012), who in virtue of a thoroughgoing belief in the indeterminacy of any notion not absolute between *any* model of first-order $\mathbf {ZFC}$, hold that we do not even have a determinate concept of natural number or ordinal. One might think then that such a view has *no* place for the iterative conception; since there is no absolute concept of ordinal we cannot iterate along the ordinal number sequence to obtain the various candidates for our $V_\alpha$. Such an argument would be too quick, however, since *any* universe in Hamkins’ ontology believes itself to have its own ‘iterative conception’ in which the sets reside (indeed, it is a theorem of first-order $\mathbf {ZFC}$ that every set belongs to some $V_\alpha$). For a Hamkinsian multiversist, however, the iterative conception has no *absolute* significance: It does not, in addition to corresponding to a particular mathematical theorem, latch on to any extra-mathematical facts (say concerning the nature of set-theoretic subject matter). In this way, we may distinguish the *mathematical* content of the iterative conception (i.e. the theorem that every set belongs to some $V_\alpha$) from the *philosophical* content (i.e. that the iterative conception tells us what the subject matter of set theory is). Since we are interested in how the iterative conception can yield different *ontological* pictures, we set aside views of Hamkins’ kind (despite its interest for the philosophy of set theory). We will, therefore, assume for the rest of the paper that we have a determinate concept of well-ordering, ordinal, and natural number, and that since we begin with the empty set and iterate along the ordinal number sequence, whatever is thereby defined is transitive and well-founded in some *absolute* sense (i.e. there is determinate sense attaching to notions of *transitivity*, *well-foundedness*, and *ordinal* independent of a particular model of first-order $\mathbf {ZFC}$). Moreover, on the assumption that we have a determinate conception of natural number, since $V_\omega$ is absolute between transitive well-founded models of $\mathbf {ZFC}$ we should hold that $V_\omega$ is the same in every universe satisfying the iterative conception in the *philosophical* sense.

Assuming the iterative conception in the philosophical sense, it is what goes on *above*$V_\omega$ where most philosophical debate concerning actualism and multiversism in set theory occurs. In particular, worries about what is guaranteed by our conceptions of the powerset operation and ordinal number sequence will result in different combinations of actualism/multiversism. The time has come to be precise about the different senses of multiversism and actualism we will examine:

By *actualism* with respect to height/width, we mean those views which hold that there are universes of set theory which cannot be extended with respect to height/width.

By *multiversism* with respect to height/width, we mean those views which hold that any universe of set theory can be extended in the relevant dimension to a new universe of set theory.

This characterisation is essentially the same as the one provided in Antos et al. (2015), with one small difference, we opt for the term ‘multiversism’ rather than ‘potentialism’. The reason for this choice is to keep our philosophical discussion manageable; potentialism refers to a wide variety of views, each of which has subtly different philosophical commitments, and we wish to isolate very specific philosophical interactions. To show this distinction, we exhibit two differences of this kind. (1.) A potentialist in the style of Linnebo (2010) may well assert that there is *just one* universe of sets, it is just that it is modally indefinite, whereas a multiversist position developed from the ideas of Zermelo (1930) (such as Isaacson 2011 or Rumfitt 2015) is likely to say that there is an unbounded sequence of universes extending each other in height. This plays out in (2.) the ways proponents of each kind of view are likely to ascribe truth values to set-theoretic sentences. To see this, suppose that there is a $V_\alpha$ containing a measurable cardinal. A Zermelian is likely to say that this statement is neither true nor false; there are perfectly good universes containing measurable cardinals (e.g. $V_\alpha$), and perfectly good universes lacking them (e.g. if $\kappa$ is the least inaccessible, then $V_\kappa$ is just such a universe).^12^ A Linnebo-style potentialist, however, is likely to say that the statement “$(\exists x)\text {Measurable}(x)$” is *true*; on Linnebo’s view the set-theoretic quantifier $(\exists x)$ should be read as $\Diamond (\exists x)$ in a modalised set theory, and “$\Diamond (\exists x) \text {Measurable}(x)$” does hold at every world.^13^ Since conceptions of truth in set theory will be important for our arguments later, we choose to focus on *multiversism*, despite the interesting questions surrounding potentialism more generally.

Though we have characterised the dimensions of height and width as separate, they can often be intimately related. For example, there are some models that cannot be extended in height to a ‘taller’ well-founded model without also being extended in width. A good example here is the Shepherdson-Cohen minimal model of set theory.^14^ This is a countable transitive model of the form $L_\alpha \models \mathbf {ZFC}$, where $\alpha$ is the least such ordinal. Small additions of height to this model (even just two extra *L*-levels) will necessarily add extra reals,^15^ assuming that we continue to move to a well-founded transitive model.

To see that this latter assumption of well-foundedness is necessary, we require some additional terminology that will prove to be useful later. A *top-extension* of a model $\mathfrak {M}$ is a model $\mathfrak {N}$ of which $\mathfrak {M}$ is a subclass and in which $\mathfrak {M}$ is a proper rank-initial segment (though it need not be the case that $\mathfrak {M} \in \mathfrak {N}$).^16^ An *end-extension* (or *transitive extension*) of a model $\mathfrak {M}$ is (by contrast) a model $\mathfrak {N}$ which not only has $\mathfrak {M}$ as a submodel, but also adds no new sets to sets already present in $\mathfrak {M}$.^17^ Note that there are top-extensions (constructed via a definable ultrapower) of countable models of $\mathbf {ZFC}$ in which there is no least new ordinal.^18^ Recall, however, that for a universe to satisfy the iterative conception in the *philosophical* sense, we required it to be transitive and well-founded. We thus require that if one universe extends another, in order to qualify as a universe it must be an end-extension. Thus, turning back to the Shepherdson-Cohen model, we can put the point about the relation between its height and width thus: it has no well-founded top-extensions.

We then obtain four views corresponding to each possible combination of actualism/multiversism in height and width^19^:

By *Radical Actualism* we mean the view that there are universes of set theory that cannot be extended in either height or width. The normal view of this kind is *Absolutism*: the view that there is a single such universe.^20^

By *Pure Width Multiversism* we mean the view that there are universes of set theory that cannot be extended in height, but that every universe can be extended in width.^21^

By *Zermelian Multiversism* we mean the view that holds that there are universes of set theory that can be extended with respect to height, but cannot be extended with respect to width.^22^

By *Skolemite Multiversism* we mean the view that any universe of sets can be extended with respect to both height and width.^23^

Our interest here will be with how these different views interact with ideas concerning maximality. In the end we will argue that comparing the Zermelian and the Skolemite with respect to certain recently proposed set-theoretic axioms reveals that the content an axiom captures is substantially dependent upon the ontological background within which one works.

One issue here, often discussed in the literature on Absolute Generality, is how a multiversist of a particular flavour could interpret quantification over *the whole* of their multiverse given that they hold that there is no ‘absolute’ set-like domain over which they quantify. There are several options here. One might hold that despite the fact that there is no absolute universe (a *metaphysical* question), this does not preclude *quantification* over all domains (a *semantic* issue). Instead, one might (as in Glanzberg 2004; Hellman 2006), take us to be always contextually restricted and provide an explanation of how we should understand quantification. There are still many options besides.^24^

Whatever the choice of account of quantification, the account of ‘*V*’ will be schematic for the Multiversist: On a given occasion of reference ‘*V*’ operates like a free variable that can be interpreted as referring to any universe of the required form, and (in the case of an extending construction) the multiverse surrounding it. Later, exactly what the ‘required form’ comes down to will be important. For the moment, we fix notation for clarity. From now on we will use a caligraphric ‘$\mathcal {V}$’ to denote universes independent of ontology, and reserve the ‘normal’ symbol ‘*V*’ for the Absolutist’s universe. In our usage then, ‘$\mathcal {V}$’ could denote a Skolemite universe just as much as it could denote *V*, and we will be specific about any constraints we put on the use of ‘$\mathcal {V}$’ within a particular argument.

A remark on terminology is important to clear up any misunderstanding. We have chosen terms for the views that will form the focus of our analysis (namely Zermelian and Skolemite Multiversism) for a number of reasons. The first is brevity, we will introduce two characters; the Zermelian and the Skolemite,^25^ each of which subscribe to the relevant positions outlined above. Each view, as we argue below, shares some features with the ideas of Zermelo and Skolem, however we do not claim that Zermelo or Skolem themselves would assent to the views in their entirety. We wish to present arguments in philosophical exploration, not historical exegesis. Nonetheless, some remarks concerning the genesis of the two views are salient in order to isolate a particular theory of set-theoretic truth to which many multiversists adhere.

Zermelian multiversism has its roots in the work of Zermelo (1930). Central to the motivations for the view are two metamathematical observations. First, that our best second-order theory of sets $\mathbf {ZFC_2}$ is only *quasi*-categorical, in that any two models of $\mathbf {ZFC_2}$ (with the full semantics) are either isomorphic or one is isomorphic to a proper initial segment of the other. This was seen by Zermelo^26^ as a failure of our thought and language to pin down a single universe of sets, rather than an unbounded sequence thereof. Second, it is through this unbounded sequence of universes that the problem of ‘proper classes’ is dissolved; any problematic ‘collection’ is simply a garden-variety set in a well-founded top-extension. So Zermelo writes:

“Scientific reactionaries and anti-mathematicians have so eagerly and lovingly appealed to the ‘ultrafinite antinomies’ in their struggle against set theory. But these are only apparent ‘contradictions’, and depend solely on confusing set theory itself, which is not categorically determined by its axioms, with individual models representing it. What appears as an ‘ultrafinite non- or super-set’ in one model is, in the succeeding model, a perfectly good , valid set with both a cardinal number and an ordinal type, and is itself a foundation stone for the construction of a new domain. To the unbounded series of Cantor ordinals there corresponds a similarly unbounded double-series of essentially different set-theoretic models, in each of which the whole classical theory is expressed.” (Zermelo 1930, p. 1233)

So we find Zermelo asserting that our thinking concerning sets, in terms of attempting to provide a categorical second-order axiomatisation that pins down (up to isomorphism) the objects of study, only succeeds in isolating varying universes $\mathcal {V}$, each of which is of the form $(V^{\mathcal {V}'}_\kappa , \in , V^{\mathcal {V}'}_{\kappa +1})$ in some well-founded top-extension $\mathcal {V}'$ (where $\kappa$ is an inaccessible cardinal). The paradoxes are thereby avoided (so the thinking goes^27^); any apparently problematic totality is a set in an extended universe. Important for seeing the distinction between the Skolemite and Zermelian, is that for the latter extensions of universes are all *proper height extensions* in that every universe is a proper initial segment of some other universe (i.e. they do not disagree, for any set *x* contained in both, on the identity of $\mathcal {P}(x)$). Indeed, it is essential to the view that we have a determinate conception of the power set operation; the quasi-categoricity theorem depends essentially on the use of the ‘full’ second-order semantics, and fails when a Henkin interpretation equivalent to a two-sorted first-order formulation is used.

The Skolemite puts no such weight on quasi-categoricity, and does not countenance the use of the full second-order semantics in interpreting second-order resources. Rather, he sees many set-theoretic notions as essentially *relative*:

“Thus, *axiomatizing set theory leads to a relativity of set-theoretic notions, and this relativity is inseparably bound up with every thoroughgoing axiomatization.*...*on an axiomatic basis higher infinities exist only in a relative sense.*” (Skolem 1922, p. 296, original emphasis)

There are several interpretations of Skolem’s arguments available.^28^ However, of interest to us will be the idea that higher infinities are only *relative*, and how this might relate to independence. One of the central techniques motivating the Skolemite position that extensions are always available is *forcing*. This technique provides us with a method of adding sets to models, and is essential in constructing the relevant models for a wide variety of independence proofs.^29^ However, forcing also enables drastic manipulation of the cardinal structure of models. In particular, for any set *x* of cardinality $\kappa$ in some universe $\mathcal {V}$, assuming that width extensions are always available, there is a forcing (known as the *Lévy Collapse*) that collapses $\kappa$ to $\omega$ in the extension $\mathcal {V}[G]$.^30^ Thus, any set can be made countable, on the assumption that we can always move to a width extension. This idea is taken up by Meadows:

“I would like to make the provocative suggestion that forcing is a kind of natural revenge or dual to Cantor’s theorem: where Cantor gives us the transfinite, forcing tears it down.” (Meadows 2015, p. 203)

As Meadows points out, though it appears that Cantor’s Theorem implies that there are absolutely uncountable sets, given width extensions this is illusory. For, given any particular infinite set *x* in a model, the cardinality of both *x* and $\mathcal {P}(x)$ can be collapsed to the countable with a forcing construction (of course, the power set of *x* in the original model will not be the same as the power set of *x* in the extension).

There are several differences between the thinking of Skolem and Meadows. In particular, Skolem was motivated by the Löwenheim-Skolem Theorems, whereas Meadows is motivated by the character of the independence phenomenon. Meadows has in mind only width extensions, but the situation is made even more acute if top-extensions are also available. Assuming that width extensions are available, the cardinality of any set *x* within some universe $\mathcal {V}$ can be collapsed to $\omega$. If we also allow top-extensions, however, we can collapse the size of *entire universes*. For, given a particular $\mathcal {V}$, we can extend in height to some $\mathcal {V}'$ such that $\mathcal {V} \in \mathcal {V}'$, and then use the Lévy Collapse over $\mathcal {V}'$ to move to a universe $\mathcal {V}'[G]$ in which $\mathcal {V}$ is countable. The Skolemite view that extensions are always available finds expression in the work of Arrigoni and Friedman:

“Since the *hyperuniverse*, the collection of all countable transitive models of $\mathbf {ZFC}$, is closed under all possible universe-creation methods, one is led to identifying the multiverse with it.” (Arrigoni and Friedman 2013, p. 85)

This encapsulates the Skolemite position we have in mind. Though any Skolemite universe $\mathcal {V}$ will take itself to have uncountable sets, since any universe can be considered to be a countable transitive model from a suitable perspective,^31^ we can think of talk about the multiverse as concerning all such models of $\mathbf {ZFC}$. Of course, as noted earlier, what we take to be ‘all’ such models will depend upon the background we fix from the start.

One salient fact for distinguishing our Skolemite from the actual views of Skolem, is the kind of upshot Skolem took from the hypothesis that any set could be made countable:

“The most important result above is that set-theoretic notions are relative....There are two reasons why I have not published anything about it until now: first, I have in the meantime been occupied with other problems; second, I believed that it was so clear that axiomatization in terms of sets was not a satisfactory ultimate foundation of mathematics that mathematicians would, for the most part, not be very much concerned with it. But in recent times I have seen to my surprise that so many mathematicians think that these axioms of set theory provide the ideal foundation for mathematics; therefore it seemed to me that the time had come to publish a critique.” (Skolem 1922, pp. 300–301)

There is a question here of whether or not Skolem was arguing against the use of set theory as a *foundation* or trying to reject it tout court.^32^ For our purposes, however, we are interested in cases where set theory *is* foundational, and we *are* engaged in trying to resolve set-theoretic independence. Why then, does our Skolemite *not* repudiate set theory as understood through $\mathbf {ZFC}$?

The answer to this question lies in how one construes set-theoretic practice. What are we doing when we investigate set theory? One answer is that we investigate the uncountable, in some absolute sense. After all, doesn’t Cantor’s Theorem teach us that there are such sets? If one is moved by this picture of set theory, then the Skolemite’s position does repudiate set theory as a discipline worthy of foundational study.

However, this is not the only way of construing set-theoretic practice. Indeed, it is unlikely to be the Skolemite’s view of set theory, given that he is immediately committed to the non-existence of absolutely uncountable sets. Instead, he is likely to construe set theory as an investigation of our combinatorial ways of thinking and study of mathematical consistency. What different combinations of mathematical objects (set-theoretically construed) are compossible? How can we construct different mathematical models from one another? These are the kinds of questions the Skolemite sees set theory as answering. Since the notion of uncountability immediately becomes model-relative for the Skolemite, the study of uncountable sets is one concerning how different set-theoretic properties interact within a model and how they change when moving between models, rather than an examination of any absolute notion of uncountability.

This view of set theory as conceptual investigation rather than the study of the uncountable absolute has ramifications for the kind of theory of truth that the Skolemite is likely to accept. In particular, he will see part of the study of set theory as what holds relative to our set concept(s).^33^ As such his theory of truth will examine what holds in *all* universes satisfying our concept(s) of set.

“Being confronted with a bewildering number of different options is a situation which we are familiar with not only in contemporary set theory. A behavior which we naturally adopt in such a situation is the following: we analyze what the possibilities are, choose among them those that under justified criteria look better than others (hence could be privileged on a priori grounds), and decide in favour of these.” (Arrigoni and Friedman 2013, p. 86)

we then say that:

“first-order properties which are true across preferred universes of the hyperuniverse are true...” (Arrigoni and Friedman 2013, p. 85)^34^

Thus, we have a characterisation of the Skolemite position on which what is true is characterised as what holds in all models satisfying our concept(s) of set.

Despite their manifold differences, a parallel is now emerging between the Skolemite and the Zermelian. Each wishes to assert that there are different, equally legitimate set-theoretic universes, and no maximal such. Each universe in their ontology satisfies the iterative conception in the philosophical sense, in that they hold there to be absolute significance to the notion of well-ordering and ordinal, and their universes are obtained by iterating along the ordinals. Truth, for each, is to be understood through analysing what holds across universes satisfying our set concept(s).^35^ The difference, however, is that they disagree on what our concept(s) of set guarantee(s) to be determinate, and hence on the nature of their respective multiverses. The Zermelian holds that our conception of the powerset operation is determinate, and that we should understand universes as models of $\mathbf {ZFC}_2$. Given a universe $\mathcal {V}$, we can view $\mathcal {V}$ as of the form $(V^{\mathcal {V}'}_{\kappa }, \in , V^{\mathcal {V}'}_{\kappa + 1})$ (for $\kappa$ strongly inaccessible) in some $\mathcal {V}'$ extending $\mathcal {V}$ in height. The Skolemite, on the other hand, regards the independence phenomenon as indicative of indeterminacy in the powerset operation as well as the ordinal number sequence. Hence, he has as universes various $\mathcal {V}$ that are countable in some extension $\mathcal {V}'$. While the ontology is radically different, the underlying conception of truth is similar. Indeed, the conception of truth is the same for the Absolutist. Truth for them is also construed as what holds across all universes satisfying our concept of set. On their picture, however, since the powerset operation *and* length of the ordinals is fully determinate, there is only one universe satisfying the concept of set in the fullest sense. *Truth* is still *truth across the multiverse*, it is just that it is a multiverse containing only one universe.^36^ This similarity in conceptions of truth will turn out to be important when we come to assess characterisations of maximality on each conception. We do not deny that there are other views of set-theoretic truth. For example, Linnebo (2010) views set-theoretic truth as an essentially *modal* phenomenon: an existential set-theoretic statement $\exists x \phi (x)$ is true just in case $\Diamond \exists x \phi (x)$ holds (and $\Box \forall x \phi (x)$ in the case of universal generalisations).^37^ In this paper, we simply restrict ourselves to multiversists who have the above conception of set-theoretic truth (e.g. on the Zermelian side Isaacson 2011; Rumfitt 2015; Antos et al. 2015, and on the Skolemite side Arrigoni and Friedman 2013). Again, we emphasise that though the views of Skolem and Zermelo have plausibly inspired much work in the philosophy of set theory, it is unclear that either Skolem or Zermelo would have assented to the conception of truth outlined here.^38^

### Maximality

Given the characterisation of actualisms and multiversisms of various kinds above, we might ask how we might go about resolving independence. One suggestion is to examine features of our concept(s) of set in trying to formulate and justify new axioms, and this is the approach we shall analyse here.^39^ A putative feature of our concept(s) of set that has been put forward is *maximality*. The thought behind such a view is that we should privilege universes which have certain maximality properties. One might hold, say, that the ordinals should be closed under certain operations in order for a universe to qualify as a bona fide universe of sets. Alternatively, one might think that a universe should contain non-constructible reals in order to be maximal. The idea has some precedent within the literature. Aside from Gödel’s earlier remark, we can find Drake saying:

“We look for justification for these axioms^40^ from the point of view of the cumulative type structure, where we want to say that the collection of levels, which is indexed by the ordinals, is a very rich structure with no conceivable end.” (Drake 1974, p. 123)

Similar remarks are to be found in Wang:

“We believe that the collection of all ordinals is very ‘long’ and each power set (of an infinite set) is very ‘thick’. Hence any axioms to such effect are in accordance with our intuitive concept.” (Wang 1984, p. 553)

Of course, it is in the meaning of the terms “very long” and “very thick” where the actualists and multiversists of various stripes will disagree with one another. For an actualist in height, the term “very long” or “as far as possible” has a single univocal interpretation; the length of the ordinal number sequence. For the Skolemite and Zermelian, on the other hand, there is no one univocal interpretation of what “very long” or “as far as possible” means, rather it will correspond to certain features of the sequence of ordinals within the particular hierarchies they countenance as satisfying the relevant maximal conception of set. Similarly, the Skolemite (as well as Meadows and Steel) will hold that there is no univocal interpretation of the term “very thick”, rather this will correspond to the existence of certain kinds of subsets available in any universe satisfying our maximal conception of set.

Maximality has received some attention, often because different scholars are more (or less) optimistic (or pessimistic) about the prospects for such a strategy.^41^ While this literature is interesting and important, our focus here is on how maximality and ontology interact. We will therefore assume for the rest of the paper that maximality represents a promising line of enquiry that we would like to capture axiomatically.

## Complementary problems

In formulating and justifying different maximality axioms, species of actualism and multiversism face complementary problems. The issue concerns the fact that often talking about extensions is useful for making maximality claims about universes.

This is true with respect to both height and width extensions. Concerning well-founded top-extensions and height maximality, the following axiom has been proposed:

**Definition 1.** (Friedman and Ternullo) $\mathfrak {M}$ satisfies the *extended reflection axiom*^42^ (henceforth ‘*ERA*’) iff $\mathfrak {M}$ has a well-founded top-extension $\mathfrak {M}'$ satisfying $\mathbf {ZFC}$ such that for all first-order formulas $\phi$ and subclasses $A \subseteq \mathfrak {M}$ belonging to $\mathfrak {M}'$, if $\phi (A)$ holds in $\mathfrak {M}'$ then $\phi (A \cap V^\mathfrak {M}_\alpha )$ holds in $V^\mathfrak {M}_\beta$ for some pair of ordinals $\alpha < \beta$ in $\mathfrak {M}$.

So, for a universe $\mathcal {V}$ to satisfy the *ERA*, it must have a $\mathbf {ZFC}$-satisfying top-extension $\mathcal {V}'$ such that if $\mathcal {V}'$ satisfies $\phi$ relative to the parameter *A*, then $\mathcal {V}$ already contains a pair of ordinals $\alpha$ and $\beta$, with $\alpha < \beta$, such that $V_\beta$ can see a level (namely $V_\alpha$) that reflects $\phi$. Effectively, $\mathcal {V}$ can already see pairs of ordinals witnessing various reflection axioms. The challenge for an actualist in height is that if she wishes to assert that the *ERA* holds of some universe $\mathcal {V}$, we have to be able to refer to top-extensions of $\mathcal {V}$. Of course this is hard to interpret for the height actualist, since there are *no* top-extensions of their $\mathcal {V}$ (or *V* in the case of the Absolutitst). Thus, without further interpretation and coding of top-extensions, the *ERA* will always come out as trivially false.

Concerning width maximality, the following two axioms make use of ‘thickenings’ of universes:

**Definition 2.** (Friedman (2006)) Let $\phi$ be a parameter-free first order sentence. $\mathfrak {M}$ satisfies the *Inner Model Hypothesis* (henceforth ‘*IMH*’) iff whenever $\phi$ holds in an inner model $I^{\mathfrak {M}^{*}}$ of an outer model $\mathfrak {M}^*$ of $\mathfrak {M}$, there is an inner model $I^\mathfrak {M}$ of $\mathfrak {M}$ that also satisfies $\phi$.

The *IMH* thus states that $\mathfrak {M}$ has a high density of inner models, in the sense that any sentence $\phi$ true in an inner model of an outer model of $\mathfrak {M}$ is already true in an inner model of $\mathfrak {M}$. In this way, $\mathfrak {M}$ has been maximised with respect to *internal consistency*; it has been maximised with respect to what can be true in inner models, given its initial structure.

There are a number of reasons to find the *IMH* interesting, not least because it maximises the satisfaction of consistent sentences within structures internal to $\mathfrak {M}$. The *IMH* is thus (if true) foundationally significant; it gives us an inner model for any sentence model-theoretically compatible with the initial structure of a $\mathcal {V}$ (or *V*), and thus serves to ensure the existence of well-founded, proper-class-sized structures in which we can do mathematics. Moreover, the principle is relatively rich in consequences, for example its normal formulation implies that the Singular Cardinal Hypothesis holds. However, it is also interesting in that versions of the *IMH* can have various *anti*-large cardinal properties (indeed some formulations of the *IMH* prove that there are no inaccessibles in $\mathfrak {M}$), whilst having a relatively high consistency strength (for instance the consistency of the *IMH* follows from the consistency of a Woodin cardinal with an inaccessible above, whilst the principle itself implies the existence of an inner model with measurable cardinals of arbitrarily large Mitchell order).^43^ This is especially interesting as the *IMH* thus provides the possibility of motivating an axiom that substantially reduces the ‘cap’^44^ on the height of the ordinals, which in turn would challenge the usual orthodoxy of obtaining determinacy axioms through the use of large cardinals.^45^

Whence the problem then for the width actualist? If she wishes to use the *IMH* as a new axiom about a universe $\mathcal {V}$, she has to examine issues concerning extensions of $\mathcal {V}$. If they ascribe *no* meaning to claims concerning extensions, then the *IMH* is utterly trivial. Under this analysis, everything true in an inner model of an outer model of $\mathcal {V}$ is also true in an inner model of $\mathcal {V}$, as either (i) the outer model is proper, does not exist, and hence nothing is true in an inner model of that proper outer model of $\mathcal {V}$, or (ii) the outer model is $\mathcal {V}$ itself, and obviously anything true in an inner model of $\mathcal {V}$ is true in an inner model of $\mathcal {V}$. Thus, in this setting, the *IMH* fails to capture its intended consequences (namely the existence of many inner models facilitated by a rich powerset operation). In particular, under the present analysis, the Zermelian will be unable to use the *IMH* to express any kind of width reflection.^46^

We have discussed how we might use extensions to directly formulate notions of reflection, both with respect to width and height. It is interesting to note that it is possible to encapsulate the large cardinal consequences of reflection properties through the use of objects known as *sharps*. We suppress technical details^47^ for readability. The key fact is that through the consideration of an object (known as a *sharp*), we can define the notion of a universe being *generated by a sharp* (or just $\sharp$-*generated*), when it is the result of successive iterations of an ultrapower construction using the sharp. A model’s being sharp-generated engenders some pleasant features. In particular, it implies that any first-order property obtainable in a well-founded top-extension of $\mathfrak {M}$ (possibly with parameters) is already reflected to an initial segment of $\mathfrak {M}$.^48^ In this way, we are able to coalesce many reflection principles into a single property of a model. A natural axiom then would be:

**Axiom 3.***The Sharp Axiom.*$\mathcal {V}$ is sharp-generated.

which would allow us to assert in one fell swoop that $\mathcal {V}$ satisfies many reflection axioms (rather than having to assert them in a piecemeal fashion). Indeed, the *ERA* is itself a consequence of The Sharp Axiom.^49^ Importantly, in order for a universe to be generated by a sharp, it cannot contain the sharp from which it arises. Thus, such an axiom is clearly problematic; claiming that $\mathcal {V}$ is sharp-generated depends upon the existence of a sharp for $\mathcal {V}$, which cannot be in $\mathcal {V}$ by design for a width actualist. We then have the unwelcome result for those that might wish to use $\sharp$-generation that the claim that $\mathcal {V}$ is sharp-generated comes out as trivially false; there simply is no such sharp.^50^

So, it seems that for actualists of various stripes there are problems with formulating certain maximality axioms. For certain recently proposed axioms of set theory, it seems that we *need* extensions to formulate the axiom in a way that captures the maximality properties we intend. Of course, this might make the relevant actualist hesitant to examine such axioms. As we will show later, some actualists have the possibility of coding these axioms, and thereby have the opportunity (should they wish to take it) to examine multiple foundational programmes. However, as we shall also see, in doing so the content of the axiom shifts according to ontological view.

This might lead one to think that there are no problems for the Skolemite. For, he precisely *has* the extensions of the relevant dimension available in the way that the actualist does not. Whence then the problem?

The difficulty concerns the fact that these axioms are meant to be capturing *maximality* properties, but for the axioms in question there will be universes *extending* them that do *not* satisfy the axioms, despite containing *more* sets. Indeed, given any universe $\mathcal {V}$ in the Skolemite’s ontology satisfying one of the above axioms, there is a model in the Skolemite’s ontology extending $\mathcal {V}$ that violates exactly the same axiom.^51^ So, for different multiversists, there are axioms that purport to capture maximality that, if satisfied by some universe $\mathcal {V}$, are violated in some universes containing more sets than $\mathcal {V}$. This is puzzling; the relevant axioms were meant to be capturing *maximality*, but now there can be universes with *more* sets that violate the axioms. There are thus complementary problems at play. An actualist in a particular dimension will always have good reason to claim that a universe of the relevant kind has captured a particular kind of maximality. After all, the relevant dimension cannot be extended, and so has captured maximality of the relevant kind ‘absolutely’. However, they will be unable to use extensions in formulating maximality axioms. A multiversist, on the other hand, always has extensions available, but faces the challenge of explaining why their universes are maximal when, given some universe $\mathcal {V}$ satisfying a maximality axiom $\Phi$, there is a universe extending $\mathcal {V}$ which satisfies $\lnot \Phi$.

## Different kinds of maximality

Before providing responses, we make a remark concerning the strategy of the rest of the paper. We will now focus on a comparison of the Zermelian with the Skolemite. The reason for this, as shall be made clear, is that the possibility of coding the content of width extensions is clearer when well-founded top-extensions are available, and so we focus on views where this strategy is uncontroversial. Certainly it is an interesting question how much sense of the *ERA* can be made by the Absolutist and a multiversist of the Steel or Meadows variety. It is one, however, that we shall not address here.

### Saving the Skolemite: maximality as relational

The problem for the Skolemite is clear. Explain why a universe containing fewer sets should be more maximal than one that contains more sets. In what sense is the original universe maximal where the other is not?

A response can be obtained on behalf of the Skolemite by examining his conception of meaning and truth. Recall, for the Skolemite, that truth is determined by what holds in all universes satisfying our concept of set. Thus, the use of the term ‘*V*’ on his view is schematic; ‘*V*’ can be taken to refer to any universe of the correct form. He then has a quick response: if $\mathcal {V}'$ extends $\mathcal {V}$ but fails to satisfy the relevant maximality axiom, then it also fails to fully satisfy our concept of set.

A simple example is instructive here. Suppose that we consider some $\mathcal {V} \models ZFC$, such that $\mathcal {V}=V^{\mathcal {V}'}_\kappa$ in an extended $\mathcal {V}'$. One can ask a simplified version of the problem. Given that $V^{\mathcal {V}'}_{\kappa + 1}$ is also a perfectly legitimate mathematical object for the Skolemite, why not say that the Power Set Axiom is neither true nor false? After all, $V^{\mathcal {V}'}_{\kappa + 1}$ contains more sets than $V^{\mathcal {V}'}_\kappa$, and hence is a ‘more maximal’ model in this sense.

The answer, of course, is that $V^{\mathcal {V}'}_{\kappa + 1}$ violates our maximal concept of set in a bad way; it is part of that concept that a universe be closed under the powerset operation. Though $V^{\mathcal {V}'}_{\kappa + 1}$ is a perfectly legitimate mathematical object, it is not a *universe* in the same sense as $\mathcal {V}=V^{\mathcal {V}'}_\kappa$. The interpretation of the term ‘*V*’ to refer to $V^{\mathcal {V}'}_{\kappa + 1}$ in interpreting a set theorist would be a gross misunderstanding of the semantic content of their utterances.

So it is with universes that extend others satisfying maximality criteria for the Skolemite. On the assumption that he holds that the relevant axioms making use of extensions are good for capturing maximality in our notion of set,^52^ then the extended universes violating these axioms do not satisfy our concept of set. For the Skolemite, for a universe to satisfy a (tutored) concept of set, it must do more than merely be closed with respect to $\mathbf {ZFC}$, it must have the kinds of closure properties stipulated by the relevant maximality axioms.^53^

On the assumption that the Skolemite takes axioms involving extensions as good characterisations of maximality, this response to the problem above has profound consequences for how maximality axioms relate to our concept of set. For under this analysis, maximality is not a property held by universes *in isolation*. Rather, maximality is a property held by universes in virtue of closure properties specifiable in terms of *how they relate to other universes*. The *IMH* says that a universe $\mathcal {V}$ has been maximised with respect to internal consistency *when we take ways of expanding*$\mathcal {V}$*into account*. The *ERA* states that $\mathcal {V}$ can already see pairs of ordinals that reflect what is realisable in some *well-founded top-extension*. The Sharp Axiom states that $\mathcal {V}$ is closed under reflection properties yielded by the iteration of ultrapowers using an object *external to*$\mathcal {V}$ (namely the required sharp). Thus, for the Skolemite, maximality in our concept of set becomes a matter of how particular universes are perceived from the perspective of expanded points of view. From expanded universes, maximal universes appear saturated with satisfaction of particular kinds, and closed under particular operations, *even when the expansion is taken into account*.

### Aiding the Zermelian: maximality and infinitary proof

The problem for the Zermelian was markedly different. For her, the issue concerned the fact that she wished to make use of width extensions in stating the Sharp Axiom and the *IMH*, but did not have the extensions available. For this reason, the Sharp Axiom and the *IMH* are usually formulated as concerned with *countable* models, models which do not count as *universes* in the same sense as models of full $\mathbf {ZFC}_2$ (though they are perfectly legitimate models, they do not fully satisfy our set concept; that necessitates (at least) $\mathbf {ZFC}_2$ satisfaction).

Recent developments (especially those given in Antos et al. 2015), however, show how the content of the *IMH* and the Sharp Axiom can be coded over arbitrary *uncountable* models (such as the Zermelian’s various universes) as long as fairly mild top-extensions are available. Roughly speaking, it is possible (using an infinitary logic) to code satisfaction in outer models of uncountable structures for the Zermelian, and this facilitates formulation of the axioms over her various $\mathcal {V}$.

Before we give some details, we provide an analogy to show the broad idea. *Martin’s Axiom* is a well-known proposed axiom, and is normally formulated as follows:

**Axiom 4.***Martin’s Axiom.* Let $\kappa$ be a cardinal such that $\kappa < |\mathcal {P}(\omega )|$. For any partial order $\mathbb {P}$ in which all maximal antichains are countable (i.e. $\mathbb {P}$ has the countable chain condition), and any family $\mathcal {D}$ of dense sets of $\mathbb {P}$ such that $|\mathcal {D}| \le \kappa$, we let $MA(\kappa )$ be the claim that there is a filter *F* on $\mathbb {P}$ such that for every $D \in \mathcal {D}$, $F \cap D \not = \emptyset$. *Martin’s Axiom* is then the claim that $\forall \kappa < |\mathcal {P}(\omega )|$, $MA(\kappa )$.

Effectively, Martin’s Axiom rendered in this form states that the universe has already been saturated by forcing of a certain kind.^54^ However, we could equivalently formulate Martin’s Axiom as the following *absoluteness* principle:

**Axiom 5.***Absolute*-*MA*. We say that $\mathcal {V}$ satisfies *Absolute*-*MA* iff whenever $\mathcal {V}[G]$ is a generic extension of $\mathcal {V}$ by a partial order $\mathbb {P}$ with the countable chain condition in $\mathcal {V}$, and $\phi (x)$ is a $\Sigma _1(\mathcal {P}(\omega _1))$ formula (i.e. a first-order formula containing only parameters from $\mathcal {P}(\omega _1)$), if $\mathcal {V}[G] \models \exists x \phi (x)$ then there is a *y* in $\mathcal {V}$ such that $\phi (y)$.

The similarity between this version of Martin’s Axiom and the *IMH* is interesting; both can be viewed as principles that assert that if something is true in an extension, then it already holds in $\mathcal {V}$. The *IMH* is just more general in that it permits *arbitrary* extensions and *arbitrary* formulas (without parameters) in the form of absoluteness.

Suppose then that the Zermelian was only aware of Absolute-*MA* and not Martin’s Axiom as usually stated. Supposing that she viewed it as a natural maximality principle, could she meaningfully analyse the axiom for its truth or falsity despite its apparent reference to extensions?

The answer is clearly “Yes!”. This is because (as will be familiar to specialists) despite the fact that the Zermelian does not countenance the literal existence of the extensions, she can nonetheless capture the notion of *satisfaction in a set-generic forcing extension* using a formula (in an expanded language) that is first-order definable over $\mathcal {V}$. More specifically, by expanding our language with constants for all $\mathbb {P}$-names in $\mathcal {V}$, and closing under the usual connectives and $\in ^\mathcal {V}$, she can define a relation $\Vdash _\mathbb {P}$ (known as the *forcing relation*) in the expanded language such that: For $p \in \mathbb {P}$, if *p* were in some (‘ideal’, ‘non-existent’) $\mathbb {P}$-generic *G*, and $p \Vdash _\mathbb {P} \phi$ holds in $\mathcal {V}$, then $\mathcal {V}[G]$ would have to satisfy $\phi$ were it to exist. Moreover, if some ‘ideal’ $\mathcal {V}[G]$ were to satisfy $\phi$, then there is a $q \in G \subseteq \mathbb {P}$ such that $q \Vdash _{\mathbb {P}} \phi$.^55^ In this way, her various $\mathcal {V}$ have access to the satisfaction relation of ‘ideal’ outer models. To be clear, from the Zermelian perspective, all she is really doing here is talking about the relation $\Vdash _\mathbb {P}$ and various $q \in \mathbb {P}$ in her model, it just so happens that this talk of $\Vdash _\mathbb {P}$ mimics what would be true in extensions of $\mathcal {V}$ (were they to exist). The Zermelian can then reformulate Absolute-*MA* as follows:

**Axiom 6.***Absolute*-$MA^{\Vdash _\mathbb {P}}$. We say that $\mathcal {V}$ satisfies *Absolute*-$MA^{\Vdash _\mathbb {P}}$ iff whenever $\mathbb {P} \in \mathcal {V}$ is a partial order with the countable chain condition in $\mathcal {V}$, and $\phi (x)$ is a $\Sigma _1(\mathcal {P}(\omega _1))$ formula, if there is a $p \in \mathbb {P}$ such that $p \Vdash _\mathbb {P} \exists x \phi (x)$, then there is a *y* in $\mathcal {V}$ such that $\phi (y)$.

Thus, by coding *satisfaction* in outer models (without admitting their existence), the Zermelian can express the content of Absolute-*MA* through Absolute-$MA^{\Vdash _\mathbb {P}}$. What the Zermelian must do then, if she is to use the *IMH* and the Sharp Axiom to express anything significant, is to code satisfaction in *arbitrary* outer models, not just set-generic outer models.

Building on work of Barwise (1975), Antos et al. (2015) show how to do just this using infinitary logic. We suppress full technical details for clarity, but we can be a little more precise. We first expand our language:

**Definition 7.**$\mathscr {L}^\mathcal {V}_\in$ is the language consisting of:

(i) A predicate $\bar{\mathcal {V}}$ to denote $\mathcal {V}$.
(ii) A constant $\bar{x}$ for every $x \in \mathcal {V}$.

We can then define $\mathcal {V}$-logic:

**Definition 8.**$\mathcal {V}$-*logic* is a system in $\mathscr {L}^\mathcal {V}_\in$, with consequence relation $\vdash _\mathcal {V}$ that consists of the following axioms:

(i) $$\bar{x} \in \bar{\mathcal {V}}$$ for every $$x \in \mathcal {V}$$.
(ii) Every atomic or negated atomic sentence of $\mathscr {L}_\in \cup \{\bar{x}| x \in \mathcal {V} \}$ true in $\mathcal {V}$ is an axiom of $\mathcal {V}$-logic.
(iii) The usual axioms of first-order logic in $\mathscr {L}^\mathcal {V}_\in$.

For a set of sentences $\mathbf {T} \subseteq L^\mathcal {V}_\in$, $\mathcal {V}$-logic contains the following rules of inference:

- *Modus ponens*: From $\mathbf {T} \vdash _\mathcal {V} \phi$ and $\mathbf {T} \vdash _\mathcal {V} \phi \rightarrow \psi$ infer $\mathbf {T} \vdash _\mathcal {V} \psi$.
- *The Set-rule*: From $\mathbf {T} \vdash _\mathcal {V} \phi (\bar{b})$ for all $b \in a$ infer $\mathbf {T} \vdash _\mathcal {V} \forall x \in \bar{a} \phi (x)$.
- *The*$\mathcal {V}$-rule: From $\mathbf {T} \vdash _\mathcal {V} \phi (\bar{b})$ for all $b \in \mathcal {V}$, infer $\mathbf {T} \vdash _\mathcal {V} \forall x \in \bar{\mathcal {V}} \phi (x)$.

Proofs in this logic are then (possibly infinite) well-founded trees, with root the conclusion of the proof. Importantly, through the use of such a logic we can capture the notion of *satisfaction in an arbitrary outer model*: Consistency of theories (obtained by adding an extra predicate $\bar{\mathcal {W}}$ and the axiom that $\bar{\mathcal {W}}$ is an extension of $\mathcal {V}$ of the desired kind) in this infinitary logic codes satisfaction in an *arbitrary* outer model, just as having a $p \in \mathbb {P}$ such that $p \Vdash _\mathbb {P} \phi$ coded satisfaction in a *set-generic* outer model.^56^ Moreover, consistency in $\mathcal {V}$-logic is first-order definable in the least model of Kripke-Platek set theory containing $V^{\mathcal {V}'}_\alpha =\mathcal {V}$ (often denoted by ‘$Hyp(\mathcal {V})$’).^57^ We can then formulate the *IMH* as:

**Axiom 9.** ($IMH^{\vdash _\mathcal {V}}$) Suppose that $\phi$ is a first-order sentence. Let $\mathbf {T}$ be a $\mathcal {V}$-logic theory coding the existence of an outer model satisfying $\phi$. Then if $\mathbf {T}$ is consistent under $\vdash _\mathcal {V}$, there is an inner model of $\mathcal {V}$ satisfying $\phi$.

and the Sharp Axiom as:

**Axiom 10.***The Sharp Axiom*${}^{\vdash _\mathcal {V}}$. The theory coding the claim that there is an outer model of $\mathcal {V}$ in which $\mathcal {V}$ is sharp generated is consistent under $\vdash _V$.^58^

We defer a detailed consideration of the philosophical and technical uses of $\mathcal {V}$-logic to different work, however the philosophical point is that we can formalise what it means for a universe to satisfy either the *IMH* or Sharp Axiom in a fairly mild well-founded top-extension of a universe. We are thus able to coherently state, from the perspective of the Zermelian, what it means for a universe to satisfy these axioms.^59^

Suppose then that one is a Zermelian who views one of the *IMH* or Sharp Axiom as a good characterisation of maximality. What then is the content of these axioms? Again, they are particular ways of specifying closure properties of particular universes. However, an important *asymmetry* with the position of the Skolemite is highlighted. For under the present view, the *IMH* and Sharp Axiom are not a matter of how a universe $\mathcal {V}$ relates to other universes, but rather what is consistent in an infinitary proof system relative to their initial structure. Thus, under this conception, maximality becomes a structural feature of a universe $\mathcal {V}$ (i.e. that it permits certain $\mathcal {V}$-logic theories to be consistent), expressible in $Hyp(\mathcal {V})$, rather than a relational property of how $\mathcal {V}$ model-theoretically appears relative to other universes. While both Skolemite and Zermelian, in keeping with their view of truth as what holds across all universes satisfying our concept of set, will hold that maximality is a kind of *closure*, exactly what is captured by this closure is very different in each case. For the Skolemite, these maximality axioms fundamentally concern how a universe appears relative to others in the multiverse. For the Zermelian, maximality is a matter of how a level of richness can be ensured using consistency in infinitary proof systems.

## Conclusion and a philosophical lesson

Before we conclude, we make a short remark concerning what can be learned from the above analysis. Often in discussions of contemporary set theory, proposals for new axioms (including maximality axioms), are discussed independent of philosophical backdrop. Rather, particular formalisms are proposed and taken to express a particular maximality feature. A good example here is the ongoing discussion of whether $V \not = L$ should count as a maximising property.^60^ The above discussion challenges this methodology. What we have seen here is that background philosophical presuppositions concerning the nature of the subject matter of set theory fundamentally alter the kind of maximality being expressed by a single axiom. In one case, the *IMH* makes an assertion concerning higher-order relationships between universes, and in the another the *IMH* concerns whether or not the structure of a universe is sufficiently rich to accommodate certain properties expressed via a particular kind of infinitary logic.^61^ Thus the precise content of axioms can differ, depending on the ontological backdrop chosen. Further philosophical discussion of the justification of new axioms should pay attention not just to the axiom in isolation, but rather how the content of the axiom (and thus possibly its plausibility) can vary across different conceptions of the ontology of set theory.

In sum, maximality in set theory is a tricky subject, not least because certain proposals for new axioms involve the use of extensions in formulating notions of maximality. This creates complementary problems for multiversists and actualists of various kinds; the latter do not have the availability of extensions and the former have to contend with the existence of extensions of ‘maximal’ universes failing to satisfy the maximality criteria in question. An analysis of responses to these problems on behalf of the Skolemite and Zermelian reveals that the content of an axiom can radically differ dependent upon ontological background. Future discussion of the justification of new axioms should pay attention to this subtle feature of the semantic content of set-theoretic discourse.

## Appendix

The Appendix provides some details of technical material referred to in the text, but too lengthy to be included in footnotes.

### $\sharp$-generation

We first provide a small overview of the technical definitions of $\sharp$-generation (for details, see Friedman and Honzik 2016 and Friedman (2016)): **Definition 11.** A structure $\mathfrak {N} = (N,U)$ is called a *sharp with critical point*$\kappa$, a *sharp*, or just a $\sharp$, iff:

1. $\mathfrak {N}$ is a model of $\mathbf {ZFC}^-$ (i.e. $\mathbf {ZFC}$ with the power set axiom removed) in which $\kappa$ is the largest cardinal and is strongly inaccessible.
2. (*N*, *U*) is amenable (i.e. $x \cap U \in N$ for any $x \in N$).
3. *U* is a normal measure on $\kappa$ in (*N*, *U*).
4. $\mathfrak {N}$ is iterable in the sense that all successive ultrapowers starting with (*N*, *U*) are well-founded, providing a sequence of structures $(N_i, U_i)$ and corresponding $\Sigma _1$-elementary iteration maps $\pi _{i,j}: N_i \longrightarrow N_j$ where $(N, U) = (N_0, U_0)$.

Letting $\kappa _i = \pi _{0, i} (\kappa )$ denote the largest cardinal of the $i^{\mathrm{th}}$ iterate $N_i$, we can then use the existence of this sequence of structures $(N_i, U_i)$ and corresponding $\Sigma _1$-elementary iteration maps $\pi _{i,j}: N_i \longrightarrow N_j$ to make the following definition: **Definition 12.** (Friedman) A model $\mathfrak {M} = (M, \in )$ is *sharp-generated* (or just $\sharp$-*generated*) iff there is a sharp (*N*, *U*) and an iteration $N_0 \longrightarrow N_1 \longrightarrow N_2 ...$ such that $M = \bigcup _{\alpha \in On^\mathfrak {M}} V^{N_\alpha }_{\kappa _\alpha }$. In other words, a model is sharp-generated iff it arises through collecting together the $V^{N_i}_{\kappa _i}$ (i.e. each level indexed by the largest cardinal of the model with index *i*) resulting from the iteration of a sharp through the ordinal height of $\mathfrak {M}$.

### Violating maximality in extensions

We now provide proof sketches of how certain maximality axioms can hold in some Skolemite universe $\mathcal {V}$, but also be violated in certain extensions of $\mathcal {V}$. **Proposition 13.** Let $\mathcal {V}$ satisfy the *ERA*. Then there is a $\mathcal {V}^*$ extending $\mathcal {V}$ such that $\mathcal {V}^*$ does not satisfy the *ERA*. *Proof.* Let $\mathcal {V}^*$ be a rank-least well-founded top-extension of $\mathcal {V}$ such that $\mathcal {V}^* \models \mathbf {ZFC}$. Since $\mathcal {V}$ satisfies the *ERA*, we know that it must contain unboundedly many $V^\mathcal {V}_\alpha$ such that $V^\mathcal {V}_\alpha \models \mathbf {ZFC}$. To see this, begin by noting that $\mathcal {V}$ must have (by the *ERA*) a well-founded top-extension $\mathcal {V}'$ that sees $\mathcal {V}$ as a $V^{\mathcal {V}'}_\alpha \models \mathbf {ZFC}$, and hence $\mathcal {V}$ has a pair of ordinals $\beta$ and $\gamma$ with $\beta < \gamma$ such that $V^\mathcal {V}_\gamma$ sees that $V^\mathcal {V}_\beta$ is a model of $\mathbf {ZFC}$. However, now we note that as $\mathcal {V}'$ can see *two* rank-initial models of $\mathbf {ZFC}$ (namely $\mathcal {V}$ and $V^\mathcal {V}_\beta$), $\mathcal {V}$ has a pair of ordinals $\delta < \zeta$ such that $V^\mathcal {V}_\zeta$ sees that $V^\mathcal {V}_\delta$ sees two rank-initial models of $\mathbf {ZFC}$. Repeating this for any particular $\theta \in \mathcal {V}$, we see that if $\mathcal {V}$ contains a $\theta$-sequence of $V^\mathcal {V}_\alpha$ modelling $\mathbf {ZFC}$, then it also contains a $(\theta +1)$-sequence of $V^\mathcal {V}_\alpha$ modelling $\mathbf {ZFC}$. Bearing in mind that for any $<Ord(\mathcal {V})$-sequence of rank-initial $\mathbf {ZFC}$ models within $\mathcal {V}$, $\mathcal {V}'$ can see a $\mathbf {ZFC}$ model containing all of them (namely $\mathcal {V}$), we know that $\mathcal {V}$ also contains the relevant $V^\mathcal {V}_\alpha$ at limits, and we thus obtain the result that $\mathcal {V}$ contains unboundedly many $V^\mathcal {V}_\alpha$ modelling $\mathbf {ZFC}$. However, $\mathcal {V}^*$ was chosen to be a rank-least well-founded top-extension of $\mathcal {V}$ modelling $\mathbf {ZFC}$, and so $Ord(\mathcal {V})+1$ bounds the $V_\alpha$ modelling $\mathbf {ZFC}$ in $\mathcal {V}^*$ (and hence $\mathcal {V}^*$ does not satisfy the *ERA*). $\square$ **Proposition 14.** Let $\mathcal {V}$ be sharp generated. Then there is a $\mathcal {V}^*$ extending $\mathcal {V}$ such that $\mathcal {V}^*$ is not sharp generated. *Proof.* Since the *ERA* is a consequence of sharp generation, this follows from the previous proposition. $\square$ **Proposition 15.** Let $\mathcal {V}$ satisfy the *IMH*. Then there is a universe $\mathcal {V}^*$ extending $\mathcal {V}$ such that $\mathcal {V}^*$ does not satisfy the *IMH*. *Proof.* Again, move to a $\mathcal {V}'$ in which $\mathcal {V}$ is countable and coded by some real *R*. We then let $\mathcal {V}^*$ be a model containing *R* that satisfies $ZFC+$“Every real belongs to a countable transitive model of $\mathbf {ZFC}$”. Since the *IMH* implies that there are reals that are not in any countable transitive model, $\mathcal {V}^*$ violates the *IMH*. $\square$

## Footnotes

1 See Linnebo (2014) for an excellent survey of the literature on plural reference and quantification. Essentially, we introduce plural variables *xx*, *yy*, *zz*, etc. and quantifiers to range *plurally* over the relevant domain, so “$\exists xx \phi (xx)$” may be read as “There are some things *xx* such that $\phi (xx)$” (for a concrete example, consider “There are some apples arranged in a circle.”).

2 We provide discussion of the space of possible views in §1.3.

3 In the work of Linnebo (2010, 2013 in particular), he refers to this principle (rendered as concerned with pluralities and their modal properties) as ‘Collapse’.

4 We say *epistemic* possibility because on some conceptions of the ontology of set theory, *CH* has a truth value at this world and mathematical objects exist necessarily, and hence *CH* has a particular truth value out of necessity. On the widely held assumption that, even if such a view is true, we nonetheless do not know the truth value of *CH*, there is still a modal space of a sort for ‘possible’ values *CH* might take, where possibility involves consistency with *what we currently know*.

5 One might, for example, regard extension talk as primarily concerned with countable transitive models, as in Koellner (2013). Hamkins has his own responses to this (and other) suggestions for providing simulacra for discourse involving outer models and the symbol ‘*V*’ (see Hamkins 2012). We discuss these issues in Barton and Antos et al.

6 Of course, whether there is such a theory (or family of theories) will depend somewhat on one’s foundational tastes. We discuss this further in §3.

7 Some scholars are circumspect about the possibility of extending $\mathbf {ZFC}$ with maximality principles harmonising with the concept of set. Feferman, for example, remarks that “...it is hard to see how there could be any non-circular sharpening of the form that there as many such sets as possible.” (Feferman et al. 2000, p. 411). Others are more positive, such as Friedman (2016). The issue of whether maximality is a *good* strategy to pursue is, for present purposes, irrelevant. Here we only wish to analyse how maximality principles interact with ontology, and so shall *assume* that studying maximality in set theory is both potentially fruitful and worthwhile.

8 Gödel has in mind here the axiom that every set is constructible, otherwise known as $V=L$.

9 See, for example, Koellner (2010), Arrigoni and Friedman (2013), and Welch (2014).

10 We set aside here the thorny philosophical and metamathematical issues concerning impure sets (i.e. sets that contain non-sets as elements). See McGee (1997), Menzel (2014), and Rumfitt (2015) for some discussion.

11 See Boolos (1971) for a putative justification of $\mathbf {ZFC}$, Boolos (1989) for an expression of self-doubt about what iterativity guarantees, while Parsons (1977) worries about the interpretation of the iterative conception, and Paseau (2007) analyses putative justifications.

12 Both Isaacson (2011) and Rumfitt (2015) express this sentiment with respect to large cardinals, but also possibly when concerned with certain axioms that are ‘unbounded’ in their claims, for example the *Generalised* Continuum Hypothesis. For example, if the *GCH* held up to some inaccessible $\kappa$ but failed above, it would be neither true not false.

13 Here we assume that the Linnebo-style potentialist is an actualist in width, since if they were not, the measurability of the relevant cardinal could be destroyed in a width extension. Given the focus on plural logic in Linnebo’s work, this is a natural assumption, however it is one that could be modified and the relevant form of potentialism (in both height and width) studied.

14 See Shepherdson (1951, 1952, 1953), and Cohen (1963).

15 To see this, note that in a model $L_\beta$ of $V=L$, first-order $\phi$ is true iff for some *n*, $\phi$ is $\Sigma _n$ and there exists a satisfaction predicate for $\Sigma _n$ formulas which says that $\phi$ is true. These partial satisfaction predicates range over $L_{\beta +1}$ (i.e. are $L_\beta$-definable) and thus this yields a satisfaction predicate for $L_\beta$ which is first-order definable over $L_{\beta +1}$ (and therefore belongs to $L_{\beta +2}$). Since every set is definable in the Shepherdson-Cohen minimal model (let it be denoted by ‘$L_\alpha$’) this satisfaction predicate appears as a real in $L_{\alpha + 2}$, and so any addition of height to another well-founded model of $\mathbf {ZFC}$ will necessarily add reals.

16 More formally: (i) $\mathfrak {M}$ is a proper submodel of $\mathfrak {N}$ and (ii) whenever $a \in N / M$ (i.e. *a* is in the difference between the two domains of the two models) and $b \in M$, then *a* has higher rank in $\mathfrak {N}$ than *b* does in $\mathfrak {N}$.

17 More formally: If $a \in ^\mathfrak {N} b \in M$ then $a \in M$.

18 See Chap. 4, §4 of Chang and Keisler (1990) for details of the construction, and Fuchs et al. for a recent application.

19 Again, this way of characterising the distinction largely mirrors that of Antos et al. (2015).

20 See Gödel (1964) and Welch (2014) for views of this kind. Actualism has a variety of meanings in the literature, for example Linnebo (2013) uses the term ‘actualist’ to refer to the position we call ‘Absolutism’. This is tempered by the fact that in Linnebo (2013) (and other work, such as Linnebo 2010), Linnebo uses the term ‘*actual* world’ to refer to a particular stage in the construction of the (inherently potential) hierarchy of sets. In order to avoid confusion, we emphasise the following: we are merely fixing *our* usage of the term here.

21 See here Steel (2014) and Meadows (2015). The issues in Steel (2014), however, are somewhat subtle; Steel chooses proper class models of $\mathbf {ZFC}$ as universes in articulating a view in which he advocates a shift in foundations to a multiverse language.

22 Pertinent examples here are Zermelo (1930), Hellman (1989), and Isaacson (2011).

23 For examples of this sort of view, see Arrigoni and Friedman (2013).

24 See Rayo and Uzquiano (2006) for a short overview of some options.

25 The Zermelian will, to avoid ambiguity, be referred to using female pronouns, whilst the Skolemite will be male.

26 See, for later developments, Hellman (1989), Isaacson (2011), and Rumfitt.

27 There is a substantial question as to how much the Zermelian avoids the paradox, after all it seems as though the sequence of universes is itself a proper class. Since our focus is on how maximality and ontology interact, we set aside this difficult issue.

28 For an excellent survey, see Bays (2014).

29 We suppress the details of forcing for philosophical clarity. The interested reader is directed to Kunen (2013).

30 See Kunen (2013) and Jech (2002) for details.

31 Of course, the same universe will also be *uncountable* from a different perspective e.g. itself.

32 See Bays (2014) for discussion and references.

33 We say “concept(s)” rather than “concept”, as we remain neutral on the possibility of divergent concepts of set for the Skolemite.

34 Though Arrigoni and Friedman refer to first-order properties here, in Arrigoni and Friedman (2013) they explicitly consider them as consequences of higher-order axioms. We shall see some discussion of these kinds of axioms in later sections.

35
Koellner (2013) refers to this as “the *multiverse conception of truth*”. Both our Skolemite and Zermelian would count as *relative broad multiverse conceptions* in his sense.

36
Koellner (2013) refers to this view as the ‘Narrow Multiverse’: “the conception where the multiverse consists of one element, namely *V*”.

37 The views in Hellman (1989) are very similar.

38 Certainly Skolem seems to be arguing against the use of set theory as a foundation (though the exact interpretation is unclear), and Zermelo is more focused on providing an axiomatisation of structures (and possibly also resolving paradox). Neither is clearly concerned with set-theoretic truth.

39 Certainly Gödel (1964) is optimistic about such a strategy. For a pessimistic voice, see Maddy (2011).

40 Drake has in mind here reflection principles.

41 For some salient discussion, see Koellner (2009), Welch (2014), Friedman (2016) and Barton (2016).

42 Friedman and Ternullo in fact use the term ‘ordinal maximality of $\mathfrak {M}$’ instead of ‘$\mathfrak {M}$ satisfying the extended reflection axiom’ largely because (Friedman and Ternullo) is concerned with *maximality criteria* on universes. As we are interested in *axiom formulation*, we opt for the term ‘extended reflection axiom’.

43 See Friedman (2006) for the technical details.

44 Talk of a ‘cap’ on the ordinals is somewhat difficult, as usually the term is taken to talk about properties of *cardinals* that cannot exist. Thus, the term ‘cap’ denotes a relationship between height and width, rather than only height. For example, one can have countable models with a highly impoverished conception of the power set operation that believe they contain supercompact cardinals. For this reason, even assuming a definite power set operation (and hence fixing of this aspect of the cardinal properties of *V*), what one takes to be the cap will depend on other properties of *V*. If $V=L$ (and there are no width extensions of *V*), the cap appears as early as $0^\sharp$. Assuming *AC*, there cannot be a Reinhardt cardinal (i.e. there is no non-trivial elementary $j: V \longrightarrow V$). The point here is that the *IMH* pulls this cap all the way down to one of the *smallest* kinds of large cardinal. For a detailed discussion, see Arrigoni and Friedman (2012).

45 For a survey of this literature, see Koellner (2011).

46 We shall see a method of responding to this worry in §3.

47 We direct the reader interested in the details to Friedman (2016) and Friedman and Honzik (2016), and provide the technical details in the Appendix.

48 See Friedman (2016) and Friedman and Honzik (2016) for discussion.

49 See Friedman and Honzik (2016) for the details of the proof.

50 In the next section, we shall see how the width actualist (using work from Antos et al. 2015) can respond to this difficulty.

51 For the interested reader, we provide proof sketches in an Appendix.

52 This is a substantial assumption; the maximality axioms on offer are many and varied. We simply wish to present the *IMH* and Sharp Axiom as case studies in how maximality, axiomatisation, and ontology interact.

53 In the case of the *ERA*, *IMH*, and Sharp Axiom, a substantial technical difficulty is how to effectively mesh these principles (versions of the *IMH* are inconsistent with the Sharp Axiom and the *ERA*). The interested reader is directed to Friedman (2006), Friedman and Honzik (2016), and Friedman (2016) for details and discussion.

54 The same goes for other forcing axioms such as the *Proper Forcing Axiom*.

55 See Kunen (2013) for details.

56 We defer detailed philosophical and technical consideration of $\mathcal {V}$-logic and its applications to Antos et al.

57 See Barwise (1975) or Antos et al. for details.

58 The issues are subtle here, as a formulation of full $\sharp$-generation depends upon a whole sequence of iterations, and hence requires quantification over many $\mathcal {V}$-logic theories. What we have actually formulated here is an axiom of *weak*$\sharp$-generation. We direct the reader interested in the technical details of formulating full $\sharp$-generation to Friedman.

59 A salient and interesting issue here concerns what resources an Absolutist (or certain kinds of Height Actualist) requires to express these axioms: Thus far we have required the availability of certain top-extensions in using $Hyp(\mathcal {V})$. As it turns out, the question is intimately linked to what amount of Class Comprehension they allow, see Antos et al. for details.

60 For discussion, see Maddy (1998, 2011).

61 It should be noted here, that the Skolemite can also express the *IMH* in terms of infinitary proof systems, as he also has top-extensions available. Here, we should note that since each universe can be made countable, the relevant completeness theorem holds, and so the two formulations become equivalent (see Barwise 1975). This is not so for the Zermelian, where the structures in question are absolutely uncountable, and thus the relevant completeness theorem fails for $\Sigma _1$ formulas.
